# CAR-γδ T Cells Targeting Claudin18.2 Show Superior Cytotoxicity Against Solid Tumor Compared to Traditional CAR-αβ T Cells

**DOI:** 10.3390/cancers17060998

**Published:** 2025-03-17

**Authors:** Yueqi Zhao, Yinghui Li, Shuaiqi Wang, Jingyi Han, Mingyang Lu, Yupeng Xu, Wenhua Qiao, Menghua Cai, Yi Xu, Yu Hu, Jianmin Zhang, Hui Chen, Wei He

**Affiliations:** 1 Department of Immunology, CAMS Key Laboratory T-Cell and Cancer Immunotherapy, Institute of Basic Medical Sciences, Chinese Academy of Medical Sciences and School of Basic Medicine, Peking Union Medical College, State Key Laboratory of Common Mechanism Research for Major Diseases, Beijing 100005, China; s2022005053@student.pumc.edu.cn (Y.Z.); lyhui926@pumc.edu.cn (Y.L.); b2023005058@pumc.edu.cn (S.W.); b2022005055@student.pumc.edu.cn (M.L.); b2022005063@student.pumc.edu.cn (Y.X.); 18717598717@163.com (W.Q.); menghuacai@ibms.pumc.edu.cn (M.C.); xuyi@ibms.pumc.edu.cn (Y.X.); huyu@ibms.pumc.edu.cn (Y.H.); zhangjianmin@ibms.pumc.edu.cn (J.Z.); 2 Department of Thoracic Surgery, Qilu Hospital of Shandong University, Jinan 250012, China; 202462000588@email.sdu.edu.cn; 3 Changzhou Xitaihu Institute for Frontier Technology of Cell Therapy, Changzhou 213000, China; 4 Beijing Jiadehe Cell Therapy Technology Co., Ltd., Beijing 100176, China

**Keywords:** γδ T cell, CLDN18.2, universal CAR-T cell, immunotherapy

## Abstract

Background: Claudin18.2 (CLDN18.2) is highly expressed during the development of various malignant tumors, especially gastric cancer, and CAR-T cells targeting CLDN18.2 have therapeutic potential. However, their dependence on the major histocompatibility complex (MHC) for antigen recognition limits their application. Human Gamma Delta (γδ) T cells, with strong MHC-independent cytotoxicity to most solid tumors both in vivo and in vitro, are emerging as ideal cells for the generation of robust universal CLDN18.2 CAR-T cells to treat solid tumors. Our aim was to construct a universal CAR-γδ T cell targeting CLDN18.2. Methods: We constructed novel CAR-CLDN18.2-γδ T cells by lentiviral infection and compared their superior efficacy in the treatment of CLDN18.2-positive solid tumors in vivo and in vitro. Results: CD3ζ expression was verified in HEK293T cells after lentiviral transfection of CLDN18.2 CAR, and the lentivirus was packaged and concentrated to a titer of 4.90 × 10^8^ TU/mL. Primary γδ T cells and αβ T cells were infected with efficiencies of approximately 31.76 ± 4.122% and 44.13 ± 4.436%, respectively. CAR-CLDN18.2-γδ T cells exhibited specific cytotoxicity against CLDN18.2-positive gastric cancer cells and secreted relatively high levels of Granzyme-B, Perforin-1, and IFN-γ. CAR-γδ T cells also showed superior cytotoxicity to target cells compared to classical CAR-αβ T cells in vitro. Finally, the antitumor activity of γδ T-CAR-CLDN18.2 cells was evaluated in tumor-bearing NSG mice, and CAR-CLDN18.2-γδ T cells significantly inhibited tumor growth and prolonged the survival of the mice. Conclusions: Our results demonstrate that universal CAR-CLDN18.2-γδ T cell is promising for the treatment of CLDN18.2-positive solid tumor and provide insights for the development of more universal CAR-γδ T-cell strategies for tumor immunotherapy.

## Simple Summary

Classic CAR-αβ T cells are a milestone in cancer immunotherapy. However, αβ T cells have a poor invasion of solid tumors, and the recognition of antigens is limited by a major histocompatibility complex, which is limited to autologous therapy. γδ T cells recognize antigens independent of the presentation of major histocompatibility complex molecules and can be used for allogeneic therapy. In this study, we designed a novel type of CAR-γδ T cell targeting Claudin18.2 by infection of primary γδ T cell with lentivirus. The CAR-CLDN18.2-γδ T cells show superior cytotoxicity against solid tumors than classical CAR-CLDN18.2-αβ T cells. Our results provide a new idea for the allogeneic CAR-T treatment strategy for CLDN18.2-positive solid cancer.

## 1. Introduction

Claudin (CLDN) family proteins are crucial for the formation of tight junctions [1,2,3]. Claudin18 (CLDN18) is a membrane-bound protein that is composed of two extracellular loops, four transmembrane domains, and one intracellular domain and forms tight junctions between epithelial cells, playing an important role in maintaining cell polarity and barrier function [1,2,4,5]. The first exon of CLDN18 can be selectively spliced to form two distinct splicing mutants, CLDN18.1 and CLDN18.2, with highly homologous amino acid sequences [6,7]. CLDN18.2 is highly expressed in normal gastric tissues [8], lowly expressed in normal healthy tissues, and not expressed in undifferentiated gastric stem cells [1]. CLDN18.2 is usually localized in the tight junctions of gastric mucosal cells [2,4,5,9] and plays a role in maintaining the barrier function of the gastric mucosa to prevent the leakage of gastric acid through the paracellular pathway [6]. Previous studies [8] have revealed that CLDN18.2 is involved in malignant transformation and is highly expressed in primary gastric cancer and its metastases. Aberrant expression of CLDN18.2 is also frequently found in pancreatic, esophageal, ovarian, and lung tumors [8,10,11,12]. As a result, interest in CLDN18.2 as a therapeutic target has increased. At present, tumor immunotherapy drugs targeting CLDN18.2 mainly include monoclonal antibodies [13,14,15], bispecific antibodies (BsAbs) [16,17], chimeric antigen receptor T (CAR-T) cells [18,19], and antibody-drug conjugates (ADCs) [16]. In clinical trials of CAR-T cells targeting CLDN18.2, patients exhibited obvious cytokine release syndrome and hematological toxicity, demonstrating that this treatment needs further study before it can be safely administered to patients [18]. In addition, since the recognition of antigens by classical CAR-αβ T cells requires the presentation of MHC molecules, these cells cannot be used for allogeneic therapy, which greatly limits the clinical application of CAR-T-cell therapy.

γδ T cells are a class of innate immune-like T cells that mainly consist of Vδ1 and Vδ2 cells, and Vδ2 T cells are distributed mainly in the peripheral blood [20,21,22]. The number of γδ T cells in the peripheral blood and secondary lymphatic organs is small, but they are concentrated in the skin and mucous membranes of the digestive tract, respiratory tract, and reproductive tract, in which the incidence of solid tumors is high [23,24,25]. Studies [26,27] have shown that γδ T cells infiltrating tumor tissues can directly recognize tumor-associated antigens in an MHC-independent manner, resulting in the rapid and direct killing of a variety of tumor cells. Moreover, they can also secrete a variety of antitumor cytokines, which activate other immune cells and promote an antitumor immune response; thus, the number of γδ T cells is positively correlated with the prognosis of patients with cancer [27]. Compared with the challenges encountered by many adoptive cell therapies, γδ T cells have no inherent limitations and can be sourced from healthy donors. Moreover, they can be expanded relatively easily in culture and have a powerful in vitro killing function; thus, γδ T cells are considered one of the best candidates for cancer immunotherapy [28,29,30,31]. There are currently two employed therapies that target γδ T cells. One is the selective amplification of γδ T cells in vivo by antibodies or bisphosphonate antigens. The other method is adoptive cell therapy, which involves expanding T cells in vitro or receiving an allogeneic γδ T-cell product that includes both human-derived γδ T cells and some genetically engineered γδ T cells. As of the end of 2023, a total of 47 clinical trials related to γδ T-cell immunotherapy, which are committed to the development and application of allogeneic natural γδ T cells and genetically engineered γδ T-cell products, have been registered on ClinicalTrials, and many candidate γδ T-cell therapies have achieved positive progress and clinical efficacy. γδ T cells have developed rapidly and are expected to become ideal cells for the generation of universal CAR-T cells [28,32,33].

CLDN18.2 is consistently and specifically expressed in gastric cancer and gastric metastases, and γδ T cells have unique advantages in tumor immunotherapy [5,34,35]. We hope to design a novel and universal CAR-γδ T-cell that targets CLDN18.2 for allogeneic therapy that has high invasiveness and therapeutic efficacy and is safe for patients, providing new strategies for the treatment of gastric cancer. Here, we designed a CAR-CLDN18.2 plasmid for packaging and concentration of lentiviruses. Concentrated lentivirus-infected primary T cells were used to generate novel CAR-γδ T cells targeting CLDN18.2, and the therapeutic effects of the CAR-γδ T cells were compared with those of classical CAR-αβ T cells in vitro and in vivo.

## 2. Materials and Methods

### 2.1. Main Antibodies and Reagents

The following antibodies were used in the study: APC anti-human TCR α/β antibody (Biolegend, San Diego, CA, USA, 1:100), APC anti-human TCR γ/δ (Biolegend, San Diego, CA, USA, 1:100), human CLDN18.2 antibody (Thermo Fischer, Carlsbad, CA, USA, 1:100), rabbit CD3ζ antibody (Abcam, Cambridge, UK, 1:2000), rabbit anti-GFP (Abcam, Cambridge, UK, 1:1000), mouse monoclonal β-actin (BOSTER, Wuhan, China, 1:1000), Horseradish peroxidase (HRP)-labeled goat anti-mouse and goat anti-rabbit antibodies (ZSGB, Beijing, China, 1:5000), TCR PAN γδ UNLB (Beckman, Brea, CA, USA), and Trypan blue stain (Gibco, San Francisco, CA, USA).

### 2.2. Construction of Plasmids and Production of Lentiviruses

**Figure 1:**
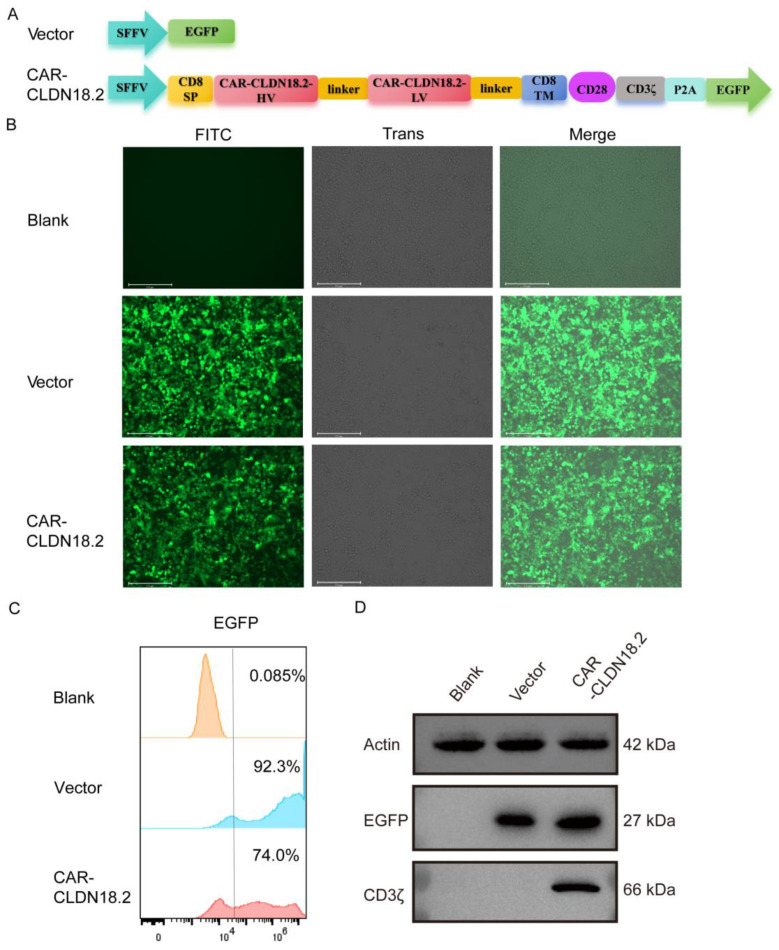
Construction and validation of CAR-CLDN18.2 expression in HEK293T cells. (**A**) Structural diagram of CAR-CLDN18.2. A vector expressing only EGFP was used as a control. SP, signal peptide; TM, transmembrane domain. (**B**) The fluorescence intensity was detected after transient transfection of HEK293T cells with the control vector or CAR-CLDN18.2 for 30 h. The scale bar is 275 μm. (**C**) The transfection efficiency of HEK293T cells was measured 30 h after transfection with the control vector or CAR-CLDN18.2 by C-flow cytometry. (**D**) The expression of EGFP and CD3ζ in HEK293T cells after transient transfection with the control vector or CAR-CLDN18.2 was detected by Western blot.

A CAR-CLDN18.2 lentivirus recombinant plasmid and a vector expressing only the EGFP fluorescent protein as a control were constructed in this study. The structure of the plasmid is shown in Figure 1A. The psPAX2 and pMD2.G plasmids were constructed as the packaging plasmid and envelope plasmid of the three-plasmid lentivirus packaging system, respectively, and were stored in our laboratory. For lentivirus packaging, the recombinant lentivirus plasmid was cotransfected with the packaging plasmids psPAX2 and pMD2.G at a 4:3:2 ratio in HEK293T cells, while the vector group lentivirus was packaged as a control. After 48 h of transfection, the cell culture supernatant was collected for ultrafiltration, and HEK293T cells were infected with different concentrations of lentivirus to detect virus titers for subsequent use. The lentiviruses were stored at −80 °C to avoid repeated freezing and thawing.

### 2.3. Cell Culture

The lung cancer cell lines A549, NCI-H520, NCI-H446, and NCI-H1299 and the gastric cancer cell lines AGS, HGC-27, NCI-N87, and HEK293T were all purchased from the Cell Center of the Chinese Academy of Medical Sciences. The gastric cancer cell line SNU-601 was purchased from Nanjing Kebai Biotechnology Co., Ltd. (Nanjing, China). SNU-601 cells stably expressing luciferase were constructed by Hefei Wanwu Biotechnology Co., Ltd. (Hefei, China). The above cells were all preserved in our laboratory. HEK293T cells were cultured in DMEM (Gibco, San Francisco, CA, USA) containing 10% fetal bovine serum, AGS cells were cultured in DF-12 (Gibco, San Francisco, CA, USA) medium containing 10% fetal bovine serum (Gibco, San Francisco, CA, USA), and the remaining cells were cultured in RPMI 1640 (Gibco, San Francisco, CA, USA) medium containing 10% fetal bovine serum. Human T cells were maintained in RMPI 1640 medium supplemented with HEPES (N-2-hydroxyethylpiperazine-N-2-ethanesulfonic acid), sodium pyruvate, nonessential amino acids, and 2-mercaptoethanol (Thermo Fischer, Carlsbad, CA, USA). The expansion of different batches of T cells also came from the peripheral blood of different healthy volunteers. All the cells were incubated at 37 °C with 5% carbon dioxide.

### 2.4. Western Blot

After transient transfection of the CAR-CLDN18.2 lentiviral recombinant plasmid into HEK293T cells for 30 h, the cells were lysed with RIPA lysis buffer, and the protein concentration was determined using a BCA assay. We used approximately 10 micrograms of protein for SDS-PAGE separation and then transferred the protein onto a nitrocellulose membrane. The samples were incubated with 5% skim milk powder at room temperature for 2 h and then incubated overnight with primary antibody at 4 °C. Then, the samples were washed three times with TBST for 5 min each, and HRP-labeled goat anti-rabbit or mouse secondary antibodies were added at room temperature for 1 h. We used the SuperSignal West Pico PLUS chemiluminescence substrate (Thermo Fischer, USA) to visualize the protein bands.

### 2.5. Flow Cytometry

An appropriate number of cells was used for detection, and corresponding controls were established. The cells were washed with PBS and blocked with 1% BSA solution. Afterward, the cells and antibodies were incubated in the dark at 4 °C for 30 min, washed with PBS, resuspended, and analyzed using a flow cytometer.

### 2.6. Expansion of T Cells In Vitro

Peripheral blood was collected from healthy volunteers, diluted 1:1 with RPMI 1640 medium, separated with lymphocyte separation medium, collected from the white blood cell layer for cleaning, and counted and plated. Microbeads coated with mouse anti-human CD3 and CD28 antibodies were added for αβ T cell expansion. To expand γδ T cells, the wells of a cell culture plate were precoated with an anti-pan-γδ antibody, and the cells were added to the wells and incubated at 37 °C for two hours.

### 2.7. Cytotoxic Activity Experiment

#### 2.7.1. Real-Time Cellular Analysis (RTCA)

The target cells were spread onto the RTCA detection plate, the detection parameters were set, and the corresponding control group was established. After the target cell index reaches a certain level, the corresponding effector cells are added. The ratio of effector cells to target cells in this experiment was 5:1. The cytotoxicity rate was calculated as follows: cytotoxicity rate = (cell index of target cell control + cell index of effector cell control − cell index of experimental)/cell index of target cell × 100.

#### 2.7.2. Lactate Dehydrogenase (LDH) Assay

The effector cells and tumor cells were cultured at a ratio of 5:1 at 37 °C for 8 h and then mixed with Promega G1780 lactate dehydrogenase detection reagent. The absorbance of each well was measured via a microplate reader at 492 nm, and no bubbles were present in the wells before measurement. The killing efficiency was calculated as follows: cytotoxicity rate = 100 × experimental LDH release amount (OD490)/maximum LDH release amount (OD490).

The target cells used in the RTCA and LDH assay were the AGS-CLDN18.2- and SNU-601-CLDN18.2+ cell lines, respectively. The effector cells used were blank-αβ T cells, Vector-αβ T cells, CAR-CLDN18.2-αβ T cells, blank-γδ T cells, Vector-γδ T cells and CAR-CLDN18.2-γδ T cells.

### 2.8. Enzyme-Linked Immunosorbent Assay (ELISA)

The supernatant from the coincubated effector cells and target cells was collected, added to the well of the ELISA plate, gently shaken evenly, sealed, and incubated at 37 °C for 2 h. After the mixture was discarded, 100 μL of biotin-labeled antibody working solution was added to each well, and the samples were sealed and incubated at 37 °C for 1 h. The liquid in each well was discarded, each well was washed 3 times with washing solution, and the mixture was aspirated for 2 min each time. Next, 100 μL of horseradish peroxidase-labeled avidin working solution was added to each well, and the plate was sealed and incubated at 37 °C for 1 h. The liquid in each well was discarded, each well was washed 5 times with washing solution, and the mixture was aspirated for 2 min each time. Next, 90 μL of TMB substrate was added to each well, and the mixture was incubated at 37 °C in the dark for 15 min. Fifty microliters of termination solution were added to each well, and the OD value of each well was measured via an ELISA reader at 450 nm within 5 min after the reaction was terminated.

### 2.9. Animal Experiments

The killing effect of effector cells on CLDN18.2-positive tumors was examined via a tumorigenicity test in NSG mice. Immunodeficient mice were randomly divided into 5 groups: a positive control group (SNU-601-CLDN18.2+), a Vector-αβ T therapy group, a CAR CLDN18.2-αβ T therapy group, a Vector-γδ T-cell therapy group, a CAR CLDN18.2-αβ T therapy group, and a CAR CLDN18.2-γδ T therapy group. Each mouse was inoculated with 5 × 10^6^ tumor cells, and treatment was started when the average tumor volume of each group reached approximately 200 mm^3^. The first cycle involved three treatments, and the second treatment cycle was carried out after one week of observation of the indicators of the mice. The second cycle involved five treatments. The αβ T cells were injected only once during the entire treatment cycle, and the γδ T cells were injected peritumorally each treatment at a concentration of 1 × 10^7^/100 μL. The body weights, tumor volumes, and other indicators of the mice were recorded regularly, and survival curves were drawn.

### 2.10. Statistical Analysis

All the representative experiments were repeated at least three times. All the statistical analyses were performed via nonpaired two-tailed *t*-tests (a *p*-value < 0.05 was considered significant, and a *p*-value < 0.01 was considered highly significant). All calculations were performed via Prism 8.0 (GraphPad) software. The data are presented as the mean ± standard error of the mean (SEM).

## 3. Results

### 3.1. Construction of CAR-CLDN18.2 γδ T Cells via Lentivirus Transfection

The third-generation CAR-CLDN18.2 molecule, which includes a CD8 transmembrane domain, a CD28 costimulatory domain and a CD3ζ intracellular signaling domain, was constructed using a lentivirus plasmid. SFFV is a strong promoter expressed in blood-derived cells [36]. The single-chain fragment variable (scFv) of a humanized monoclonal CLDN18.2 antibody was used as the core functional part of CAR-CLDN18.2, which is derived from the patent, application for publication number: CN 114751984 A. EGFP was included as a fluorescent reporter, independently expressed after P2A (Figure 1A). The expression of CAR-CLDN18.2 was first validated in HEK293T cells via fluorescence microscopy, flow cytometry, and western blot. After transient transfection with CAR-CLDN18.2 for 30 h, the HEK293T cells presented strong fluorescence signals under the EVOS fluorescent microscope (Figure 1B). Over 70% of CAR-CLDN18.2-transfected HEK293T cells positively expressed EGFP according to flow cytometry (Figure 1C). The western blot results revealed specific expression of CD3ζ at 66 kDa only in HEK293T cells transfected with CAR-CLDN18.2 (Figure 1D), indicating that the CAR-CLDN18.2 lentivirus recombinant plasmid was successfully constructed and could be used in subsequent experiments. The uncropped blots and molecular weight markers are shown in Supplementary Figure S1.

**Figure 2:**
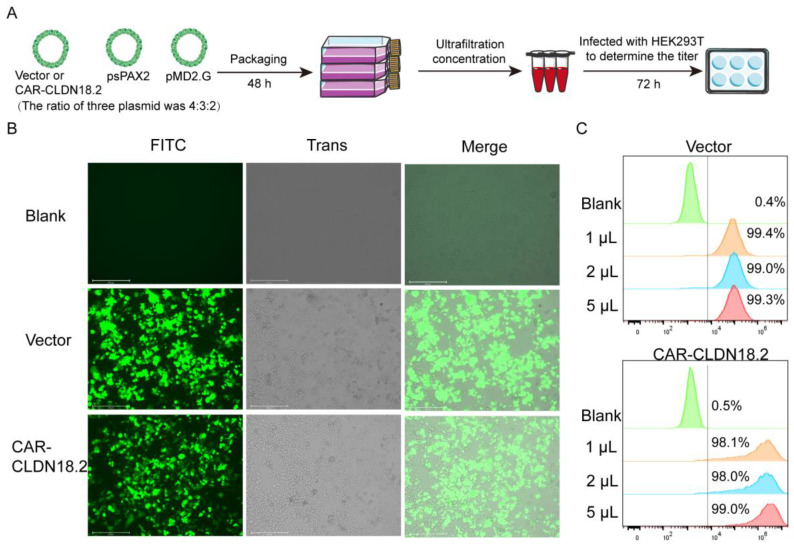
Packaging and titer determination of the CAR-CLDN18.2 lentivirus. (**A**) Flowchart. The lentivirus was transfected into HEK293T cells with CAR-CLDN18.2, the packaging plasmid psPAX2 and the envelope plasmid pMD2.G at a ratio of 4:3:2. Lentivirus was obtained by ultrafiltration of the supernatant 48 h after transfection, and lentivirus titers were detected in different volumes of HEK293T cells. (**B**) Fluorescence signals of HEK293T cells during the virus packaging procedure at 48 h. The scale bar is 275 μm. (**C**) Flow cytometry was used to determine the infection efficiency of HEK293T cells infected with lentivirus after 72 h.

A three-plasmid system was used to package the CAR-CLDN18.2 lentivirus (Figure 2A). After transfection for 48 h, the fluorescence signal of the HEK293T cells was observed (Figure 2B). The supernatant was then collected to determine the ultrafiltration concentration. On the basis of the flow cytometry data (Figure 2C), the calculated lentivirus titer of CAR-CLDN18.2 was approximately 4.90 × 10^8^ TU/mL.

**Figure 3:**
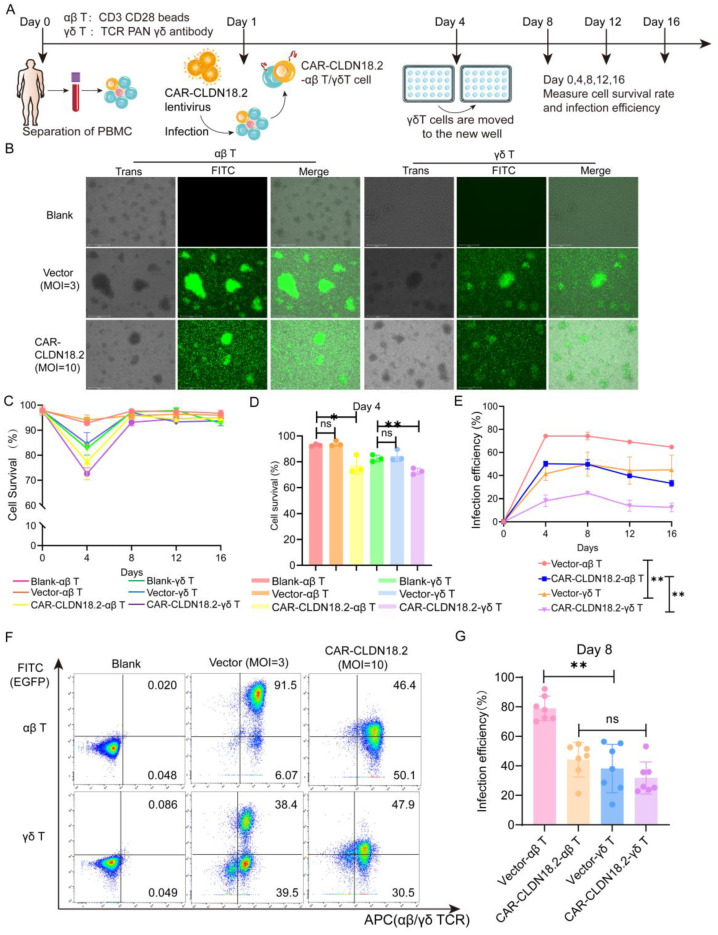
Construction of CAR-CLDN18.2-αβ T cells and CAR-CLDN18.2-γδ T cells. (**A**) Diagram of the effector cell preparation protocol. After PBMCs were isolated from the peripheral blood of healthy volunteers, they were infected with concentrated lentivirus on the first day, and γδ T cells were transferred to new wells on the fourth day. The infection efficiency and survival rate of the cells were measured every four days. (**B**) Detection of fluorescent signals of αβ T and γδ T cells after lentivirus infection. The scale bar is 275 μm. (**C**) Cell survival curve (*n* = 3). The data are presented as the means ± SEMs. (**D**) The survival rates of different effector cells were measured on day 4 (*n* = 3). The data are presented as the means ± SEMs. ns, no significant difference, *p* > 0.05; * *p* < 0.05; ** *p* < 0.01, two-tailed unpaired *t*-test. (**E**) The efficiency of vector and CAR-CLDN18.2 lentivirus infection was measured via flow cytometry at different time points (*n* = 3). The data are presented as the means ± SEMs. ** *p* < 0.01, two-way ANOVA. (**F**) Representative flow cytometry results of lentivirus infection efficiency on day 8. (**G**) Statistics on the efficiency of lentivirus infection of αβ T and γδ T cells on day 8 (*n* = 7). The data are presented as the means ± SEMs. ns, no significant difference, *p* > 0.05; ** *p* < 0.01, two-tailed unpaired *t*-test.

To evaluate the antitumor effects of CAR-CLDN18.2 γδ T cells, we simultaneously prepared CAR-CLDN18.2-αβ T cells as controls. The preparation procedure for the two CAR-CLDN18.2 T cells is shown in Figure 3A. On day 8, after the virus infection, the EGFP fluorescence signals were observed to originate from mainly the proliferated T-cell clones (Figure 3B). There was a significant decrease in the viability of both αβ T cells and γδ T cells on day 4 after CAR-CLDN18.2 lentivirus infection, and the viability gradually returned to normal after day 8, whereas the vector control seemed to have little effect (Figure 3C,D). The infection efficiency remained relatively stable after day 4, with significant differences between αβ T cells and γδ T cells (Figure 3E). The percentage of CAR-CLDN18.2-positive αβ T cells was 44.13 ± 4.436%, and the percentage of CAR-CLDN18.2-positive γδ T cells was 31.76 ± 4.122% on day 8 (Figure 3F,G). Owing to individual differences, there was no overall significant difference in infection efficiency between CAR-CLDN18.2 γδ T cells and CAR-CLDN18.2 αβ T cells. γδ T cells were derived from the peripheral blood of healthy volunteers and amplified with PAN antibodies, the subtype of which was mainly Vδ2 T cells (Supplementary Figure S2).

### 3.2. The Cytotoxic Activity of CAR-CLDN18.2 γδ T Cells Was Superior to That of CAR-αβ T Cells In Vitro

**Figure 4:**
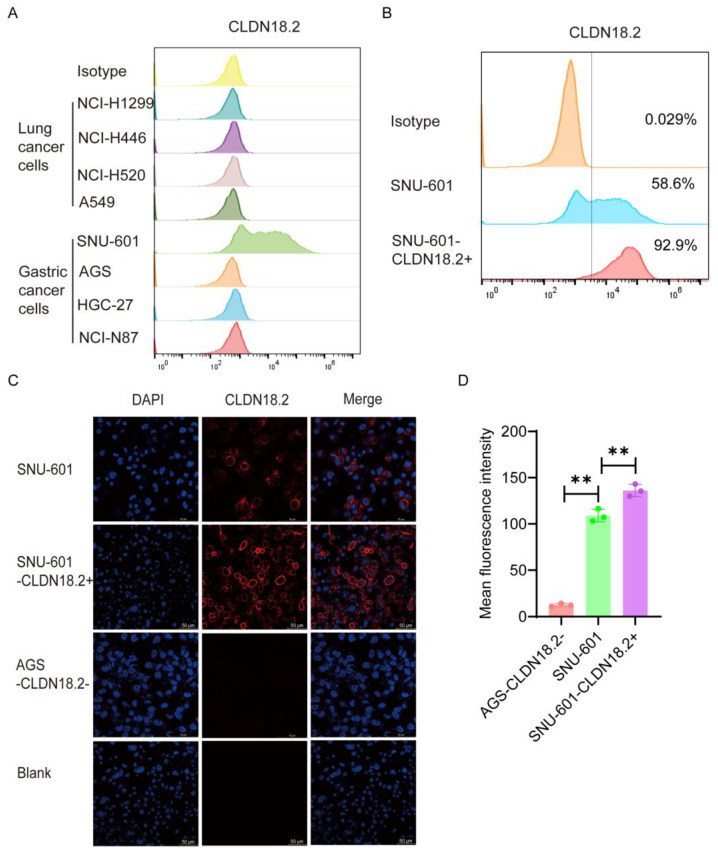
Screening and identification of target cells. (**A**) The expression of CLDN18.2 in various lung cancer and gastric cancer cell lines was detected by flow cytometry. (**B**) The expression of CLDN18.2 in SNU-601 cells before and after cell sorting was detected by flow cytometry. (**C**) The distribution and expression of CLDN18.2 (red) on SNU-601 before and after cell sorting were detected by immunofluorescence. AGS-CLDN18.2- cells were used as a negative control, and the scale bar is 50 μm. (**D**) The mean fluorescence intensity of the immunofluorescence was statistically analyzed (*n* = 3). The data are presented as the means ± SEMs, ** *p* < 0.01, according to a two-tailed unpaired *t*-test.

To verify the cytotoxicity of CAR-CLDN18.2-γδ T cells, we selected a variety of human lung cancer cell lines and gastric cancer cell lines with high expression of CLDN18.2 to be used as target cells. The flow cytometry results revealed that 58.6% of SNU-601 cells expressed CLDN18.2, whereas AGS cells were CLDN18.2-negative (Figure 4A). After cell sorting, more than 90% of cells expressed CLDN18.2 (Figure 4B), which were denoted as SNU-601-CLDN18.2+ cells. Immunofluorescence further validated the flow cytometry results. CLDN18.2 was distributed mainly on the surface of the SNU-601 cell membrane, and the fluorescence signal of CLDN18.2 significantly increased after sorting (Figure 4C,D). The human gastric cancer cell line AGS-CLDN18.2- and sorted SNU-601-CLDN18.2+ cells were used in subsequent cytotoxicity assays.

**Figure 5:**
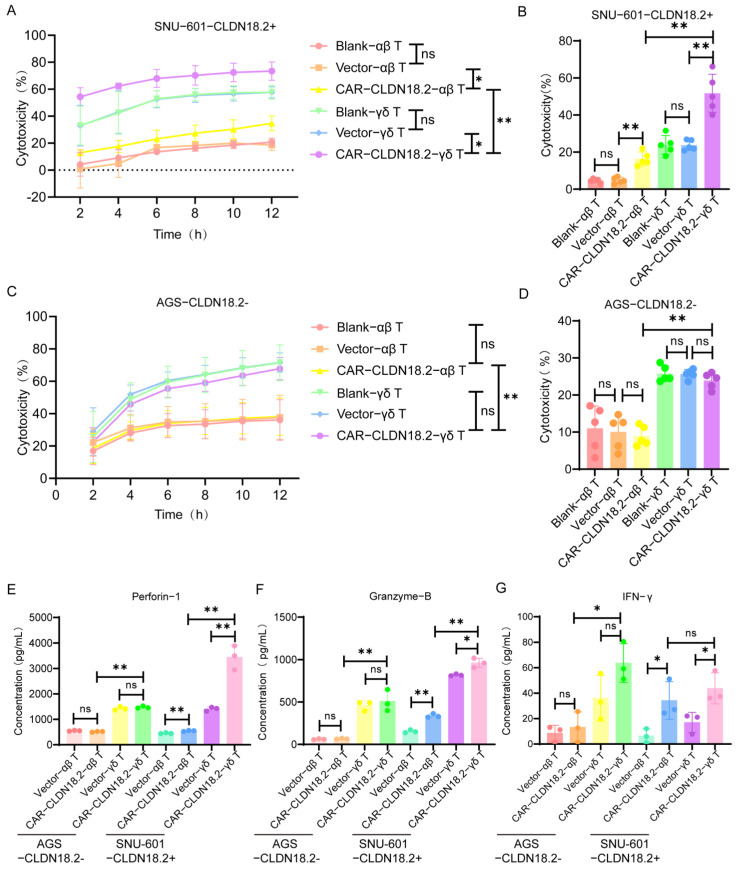
The cytotoxic activity of CAR-CLDN18.2 γδ T cells was superior to that of CAR-αβ T cells in vitro. (**A**) An RTCA was used to detect the cytotoxic activity of different effector cells against SNU-601-CLDN18.2+ cells. (**B**) The cytotoxic activity of different effector cells against SNU-601-CLDN18.2+ cells was evaluated by LDH assay. The coincubation time of the effector cells and target cells was 8 h. (**C**) The cytotoxic activity of different effector cells against AGS-CLDN18.2- cells was detected by RTCA. (**D**) LDH assay was used to detect the cytotoxic activity of AGS-CLDN18.2- toward different effector cells. The incubation time of the effector cells and target cells was 8 h. (**E**–**G**) The secretion levels of Perforin-1, Granzyme-B, and IFN-γ were measured via ELISA after different effector cells were incubated with SNU-601-CLDN18.2+ or AGS-CLDN18.2- cells for 8 h. (**A**–**G**) The ratio of effector cells to target cells was 5:1 (*n* = 3). The data are presented as the means ± SEMs. ns, no significant difference, *p* > 0.05, * *p* < 0.05, ** *p* < 0.01, by a two-tailed unpaired *t*-test.

RTCA and LDH assays were used to evaluate the cytotoxicity of CAR-CLDN18.2-γδ T cells against tumor cells in vitro. Both the CAR-CLDN18.2-γδ T cells and the CAR-CLDN18.2-αβ T cells were significantly more cytotoxic to the SNU-601-CLDN18.2+ cells than the blank and vector control cells, and these effects were sustained from 2 h to 12 h (Figure 5A). The LDH assay was used to evaluate the cytotoxicity 8 h after the coincubation of effector and target cells, and the results were consistent with the RTCA results (Figure 5B). For AGS-CLDN18.2- cells, there was no significant difference in cytotoxic activity between CAR-CLDN18.2-modified T cells and their controls (Figure 5C,D), confirming the specificity of CAR-CLDN18.2 molecules in antigen recognition. Moreover, CAR-CLDN18.2-γδ T cells presented superior cytotoxicity to CAR-CLDN18.2-αβ T cells, both for SNU-601-CLDN18.2+ cells and AGS-CLDN18.2- cells, demonstrating the powerful nonspecific tumor killing ability of γδ T cells. To compare the antitumor activity of CAR-αβ T and CAR-γδ T cells in-depth, we compared the killing activity against SNU-601-CLDN18.2+ cells by LDH at more challenging E:T ratios (1:1,1:5, and 1:10). The results showed that the antitumor function of CAR-γδ T cells was significantly higher than that of CAR-αβ T cells at the effector ratio of 1:1 and 1:5. However, under the condition of 1:10, CAR-αβ T cells could not kill tumors due to the small number of effector cells, and γδ T cells could still inhibit tumor growth (Supplementary Figure S3). Tumor cells achieve immune escape by reducing surface MHC-I molecular antigen presentation, thereby affecting the number and function of CD8+ αβ T cells mediating tumor killing [37]. In the process of co-incubation with target cells in vitro, tumor cells expressed low MHC molecules without the assistance of antigen-presenting cells, and γδ T cells still showed strong antitumor function, indicating their great potential for application in tumor immunotherapy.

The secretion of effector factors in the supernatants was measured via ELISA. Consistent with the cytotoxicity results, the levels of Granzyme-B, Perforin-1, and IFN-γ were greater in the supernatants of CLDN18.2-positive target cells treated with CAR-CLDN18.2-γδ T cells than those treated with blank and vector control cells (Figure 5E–G). CAR-CLDN18.2-γδ T cells significantly increased the secretion and expression of Granzyme-B, Perforin-1, and IFN-γ compared with CAR-CLDN18.2 αβ T cells. In addition, we detected the expression of activation markers before and after T cells were co-incubated with target cells. The expression of CD25 was higher than that of CD69, and co-incubated with target cells would affect the expression of surface activation markers (Supplementary Figure S4).

### 3.3. CAR-CLDN18.2 γδ T Cells Play a Significant Antitumor Role in Tumor-Bearing Mice

**Figure 6:**
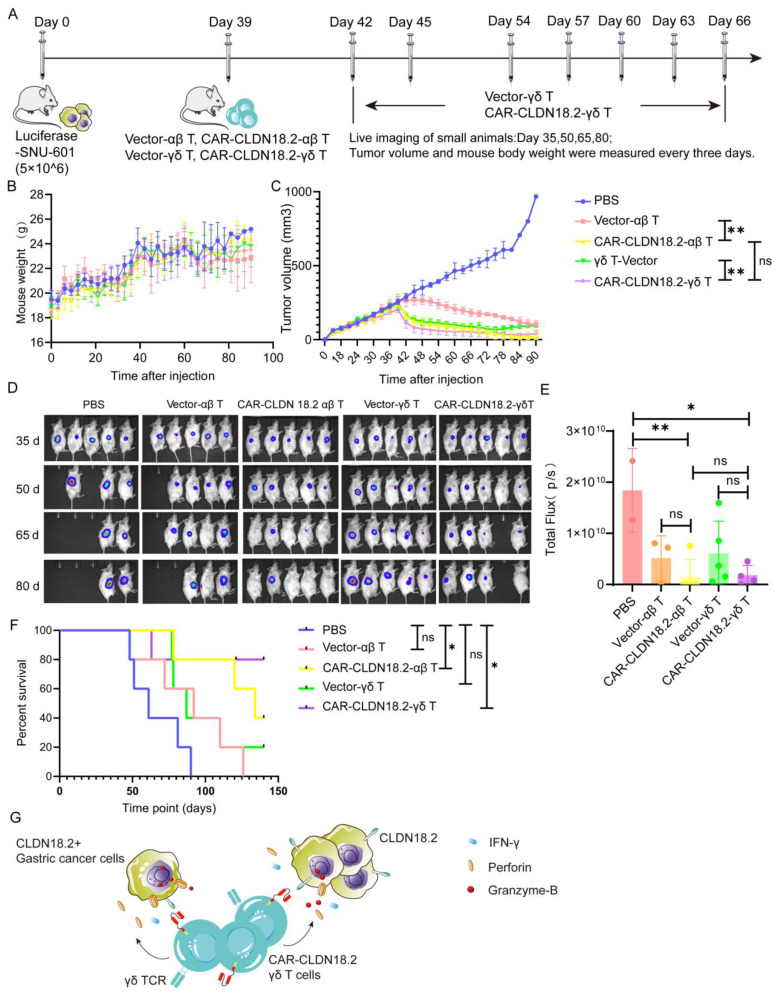
CAR-CLDN18.2-γδ T cells had an enhanced therapeutic effect on tumor-bearing mice. (**A**) Flowchart of the animal experiment protocol. A total of 5 × 10^6^ Luciferase-SNU-601-CLDN18.2+ cells were inoculated subcutaneously into immunodeficient mice. αβ T cells were inoculated once, and γδ T cells were inoculated 8 times. Each mouse was inoculated with 1 × 10^7^ cells via a peritumoral injection. On the 35th, 50th, 65th, and 80th days, tumor growth was detected via live imaging, and the weights and tumor volumes of the mice were detected every three days (*n* = 5). (**B**) Weight changes in the mice during treatment. The data are presented as the means ± SEMs. (**C**) Changes in tumor volume in the mice during treatment. The data are presented as the means ± SEMs. ns, no significant difference, *p* > 0.05, ** *p* < 0.01; two-tailed unpaired *t*-test was used. (**D**) Tumor changes in mice were detected via live imaging. (**E**) Statistical analysis of fluorescence signals via in vivo imaging after treatment. The data are presented as the means ± SEMs. ns, no significant difference, *p* > 0.05, * *p* < 0.05, ** *p* < 0.01, as determined by a two-tailed unpaired *t*-test. (**F**) Survival curve of the mice. ns, no significant difference, *p* > 0.05; * *p* < 0.05. (**G**) Theoretical diagram of the study.

To further confirm the antitumor efficacy of CAR-CLDN18.2-γδ T cells in vivo, tumor models were established in immunodeficient mice via subcutaneous injection of luciferase-SNU-601 cells and peritumoral treatment with CAR-CLDN18.2-αβ T cells or CAR-CLDN18.2-γδ T cells (Figure 6A). During the experiment, the overall weights of the mice in each group tended to increase without significant differences among the groups (Figure 6B), indicating that the cell treatment did not cause any side effects. CAR-CLDN18.2-γδ T cells and CAR-CLDN18.2-αβ T cells significantly inhibited the growth of tumors (Figure 6C–E) and prolonged the survival of tumor-bearing mice (Figure 6F) compared with PBS and vector control cells. There was no significant difference in tumor volume in mice after one dose of CAR-CLDN18.2-αβ T cells and up to eight doses of CAR-CLDN18.2-γδ T cells (Figure 6C–E). Survival data revealed that CAR-CLDN18.2 γδ T cells had slightly better efficacy than CAR-CLDN18.2 αβ T cells in vivo (Figure 6F).

Overall, our results demonstrate that CAR-CLDN18.2 γδ T cells can effectively recognize CLDN18.2-positive target tumor cells and secrete high levels of Granzyme-B, Perforin-1, and IFN-γ to exert antitumor effects (Figure 6G). Given the strong non-MHC-restricted cytotoxicity, high degree of tumor infiltration, and high safety of γδ T cells, CAR-CLDN18.2-γδ T cells are expected to become one of the most promising candidates for universal CAR-T-cell therapy of CLDN18.2-positive solid tumors.

## 4. Discussion

At present, the targeted drugs approved for the treatment of gastric cancer focus mainly on HER2, VEGFR2, PD-1, and other targets [38]. However, the expression and specificity of these targets in gastric cancer are low. The low expression of CLDN18.2 in normal tissues and its high expression in tumor tissues make it an ideal target for developing targeted therapies for solid tumors [19,39,40]. In two clinical trials, the monoclonal antibody zolbetuximab, which targets CLDN18.2, was given to patients with gastric or gastroesophageal junction adenocarcinoma, and the results revealed that zolbetuximab reduced the risk of disease progression or death by 29% [41]. A clinical trial of CT041, a CAR-T-cell-based clinical product targeting CLDN18.2, reported that the objective response rate was 57.4% and the disease control rate was 83.0% [18]. These clinical trials demonstrated the effectiveness of CLDN18.2 as a target for gastric cancer. However, obvious safety problems have been reported in clinical trials of CAR-T cells. In a phase 1 trial of CT041, all patients had grade 3 or above hematological toxicity, and 94.6% of patients developed cytokine release syndrome. In addition, CAR-T-cell recognition of antigens depends on the presentation of MHC, and these cells cannot be used for allogeneic therapy. Moreover, the infiltration of traditional CAR-T cells into solid tumors is poor [29]. These factors greatly limit the application of CAR-T-cell therapy in the treatment of solid tumors.

γδ T cells are ideal cells for the generation of CAR-T cells because they do not rely on the MHC for antigen recognition and are localized in solid tumor-prone areas such as the digestive tract [33,34,42,43,44,45]. ADI-001 [46] is a CAR-γδ T-cell therapy for B-cell lymphoma, and the objective response rate and complete response rate in clinical treatment have been found to reach 78%. Moreover, in most patients, the tumor was significantly controlled or completely disappeared after treatment. The clinical trial included several patients who relapsed after CD19 CAR-T-cell therapy and achieved a 100% complete response rate after ADI-001 treatment (NCT04735471, NCT04911478). These findings show that this therapy has a therapeutic effect on patients who relapse after CAR-T-cell therapy and provides a new treatment option for refractory patients. At the European Society of Medical Oncology (ESMO) in 2024, the phase 1 clinical trial of Unicet Biotech’s self-developed B7H3-targeting CAR-γδ T-cell drug in patients with recurrent glioblastoma (rGBM) was selected for a proffered paper session. The overall response rate was 42.9%, and the disease control rate (DCR) was 100%. Grade 3 or higher cytokine release syndrome did not occur, and graft-versus-host disease (GvHD) was not observed, indicating high safety. Our laboratory has been committed to the study of γδ T cells for a long time, and γδ T cells with genetically engineered autocrine PD-1 antibodies [47] have entered clinical trials, proving the safety of allogeneic γδ T cells. Therefore, CAR-γδ T cells have good development prospects in tumor therapy. The main limitation of the therapeutic function of γδ T cells is that they have a short half-life in vitro, cannot be steadily expanded after injection into patients, and need to be injected several times. However, due to the short survival time after injection into patients, γδ T cells are safer in clinical treatment and are not prone to immune rejection. γδ T cells have the advantage of colonizing in the skin, digestive tract, urogenital tract, and other solid tumor-prone sites, which is conducive to the migration of γδ T cells to tumor sites, and this property can also better cope with metastatic diseases [23]. Further refinement of local drug delivery methods, such as chemoembolization, catheter-directed infusion, or tumor-specific nanoparticle delivery, may provide more treatment ideas for non-epithelial metastasis. We selected severely immunodeficient mice to verify the effect of the treatment in vivo. Although their natural immune cells were close to none, it could not be completely ruled out whether there were still a small number of γδ T cells in the skin, and whether a small number of skin reserved γδ T cells would affect the therapeutic effect. In addition, the expansion efficiency of γδ T cells varies greatly among different volunteers, and screening healthy volunteers during clinical treatment is necessary to ensure the stable and effective expansion of γδ T cells. As innate immune cells, γδ T cells have a natural resistance to external pathogenic microorganisms, which increases the difficulty of successful lentiviral infection. The donor health level, γδ T-cell status, lentiviral titer, and infection conditions all affect infection efficiency.

In summary, a novel and universal CAR-CLDN18.2-γδ T-cell therapy was designed in this study, and these cells had superior cytotoxic effects compared to CAR-CLDN18.2-αβ T cells against solid tumors in vitro and in vivo because of their ability to produce high levels of Granzyme-B, perforin-1 and IFN-γ. These findings provide a theoretical basis for the use of γδ T cells in the treatment of solid tumors. Owing to their ability to recognize antigens without MHC restriction, γδ T cells are expected to become universal CAR-T cells for the treatment of solid tumors.

## 5. Conclusions

We constructed novel CAR-γδ T cells targeting CLDN18.2 by infecting primary T cells with lentivirus. Combining in vivo and in vitro experiments, the genetically engineered CAR-γδ T cells showed better cytotoxic activity than classical CAR-αβ T cells against CLDN18.2-positive solid tumors by secreting high levels of Granzyme-B, perforin-1 and IFN-γ. It is expected to be a treatment for CLDN18.2-positive solid tumors and provide insights for the development of more universal CAR-γδ T-cell strategies for tumor immunotherapy.

## Data Availability

Data supporting the present study are available from the corresponding author upon reasonable request.

## Supplementary Materials

The following supporting information can be downloaded at: https://www.mdpi.com/article/10.3390/cancers17060998/s1, Figure S1. The expression of EGFP and CD3ζ in HEK293T cells after transient transfection with the control vector or CAR-CLDN18.2 was detected by Western blot. (A) The expression of Actin was detected. (B) The expression of EGFP was detected. (C) The expression of CD3ζ was detected. Figure S2. The proportion of gd T cells amplified in peripheral blood. Peripheral blood was isolated from healthy volunteers, amplified by PAN antibody activation, and the proportion of Vδ1 and Vδ2 cells was detected by flow cytometry on day 8. Figure S3. The antitumor activity of CAR-αβ T and CAR-γδ T cells against SNU-601-CLDN18.2+ cells at more challenging E:T ratios (1:1,1:5, and 1:10) was compared by LDH method. (A) Effect-target ratio is 1:1. (B) Effect-target ratio is 1:5. (C) Effect-target ratio is 1:10. Figure S4. The changes of CD25 and CD69 were detected after co-incubation of effector cells and target cells. Different effector cells were co-incubated with negative cells AGS and positive cells SNU-601, and the expression levels of CD25 and CD69 after co-incubation were detected by flow cytometry.
