# Supplementary material for: CAR-γδ T Cells Targeting Claudin18.2 Show Superior Cytotoxicity Against Solid Tumor Compared to Traditional CAR-αβ T Cells
Source: Cancers (Basel). 2025 Mar 17;17(6):998. doi: 10.3390/cancers17060998 (PMC11940616; doi:10.3390/cancers17060998)
Supplement: Supplementary file 1 [file cancers-17-00998-s001.zip › cancers-3476363-supplementary.pdf]

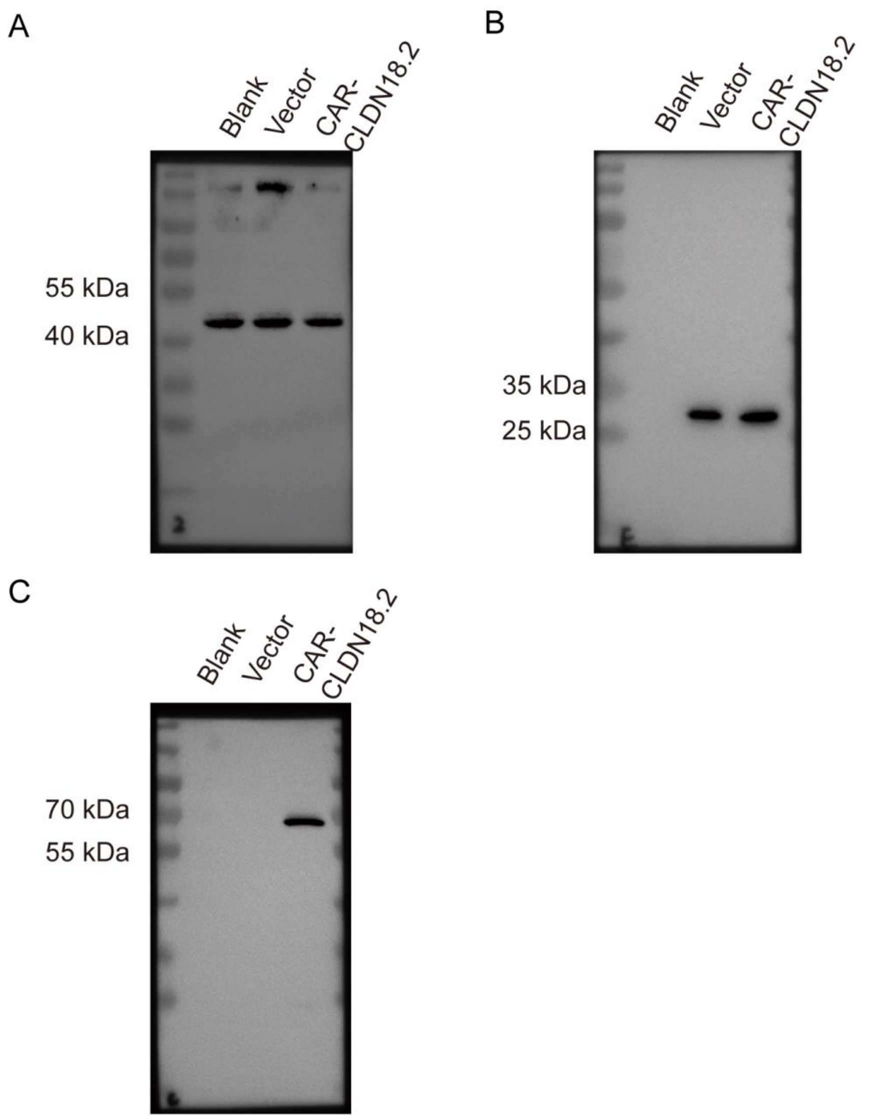

Supplementary Figure S1. The expression of EGFP and CD3ζ in HEK293T cells after transient transfection with the control vector or CAR-CLDN18.2 was detected by Western blot. **(A)** The expression of Actin was detected. **(B)** The expression of EGFP was detected. **(C)** The expression of CD3ζ was detected.

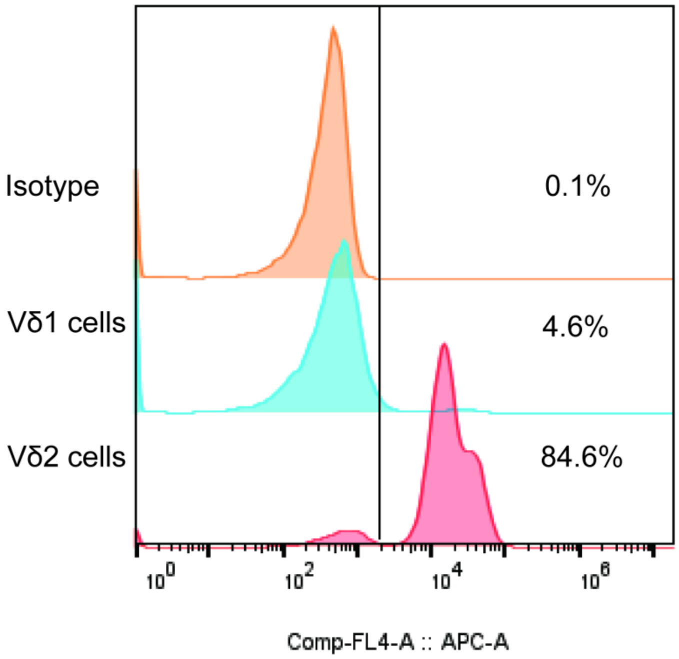

Supplementary Figure S2. The proportion of gd T cells amplified in peripheral blood. Peripheral blood was isolated from healthy volunteers, amplified by PAN antibody activation, and the proportion of Vδ1 and Vδ2 cells was detected by flow cytometry on day 8.

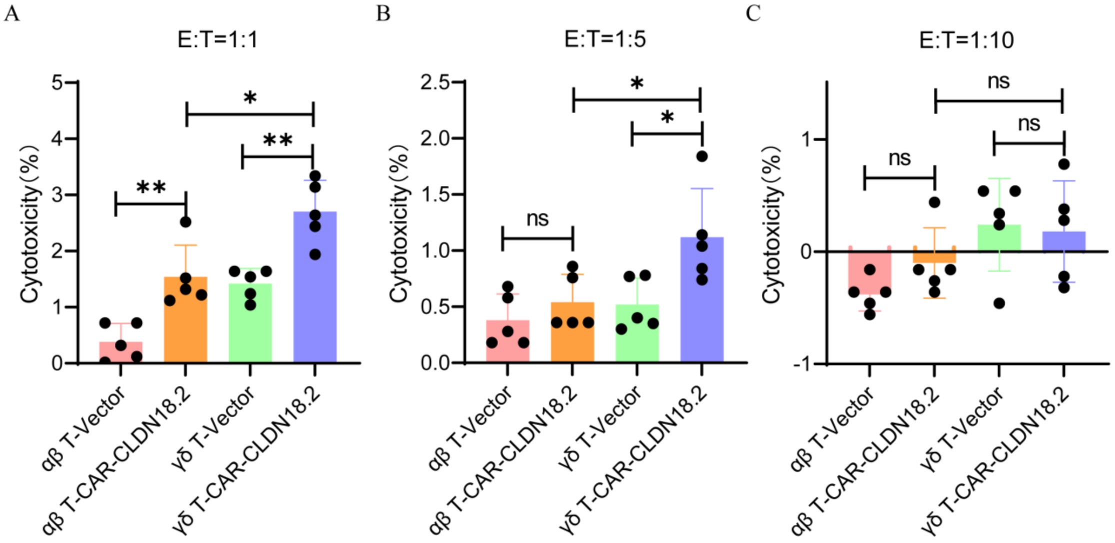

Supplementary Figure S3. The antitumor activity of CAR-αβ T and CAR-γδ T cells against SNU-601-CLDN18.2+ cells at more challenging E:T ratios (1:1,1:5, and 1:10) was compared by LDH method. **(A)** Effect-target ratio is 1:1. **(B)** Effect-target ratio is 1:5. **(C)** Effect-target ratio is 1:10.

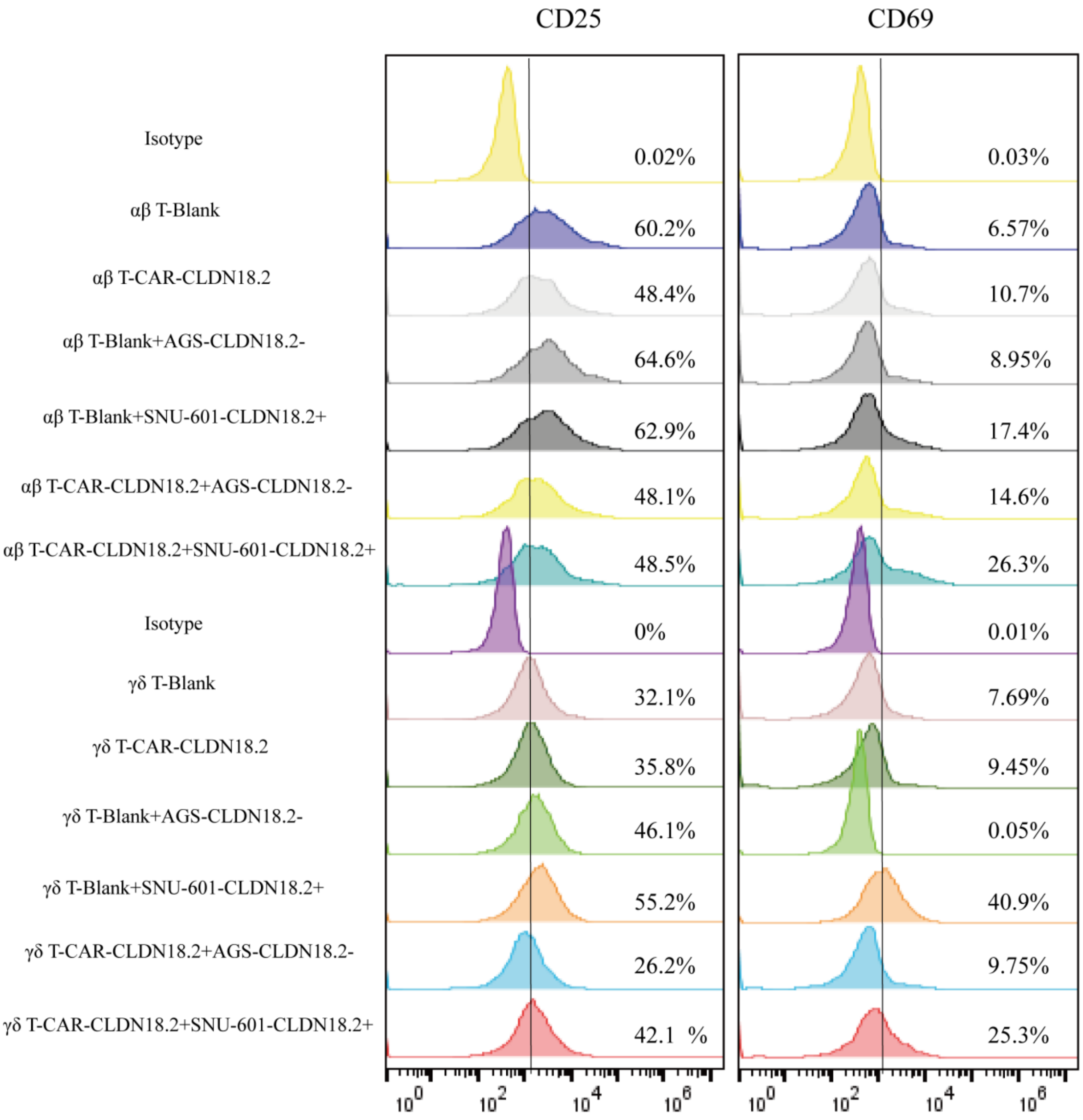

Supplementary Figure S4. The changes of CD25 and CD69 were detected after co-incubation of effector cells and target cells. Different effector cells were co-incubated with negative cells AGS and positive cells SNU-601, and the expression levels of CD25 and CD69 after co-incubation were detected by flow cytometry
